# Endovascular repair of the aortic arch

**DOI:** 10.1515/iss-2023-0029

**Published:** 2023-12-18

**Authors:** Florian Kursch, Panagiotis Doukas

**Affiliations:** Department of Vascular and Endovascular Surgery, University Hospital Cologne, Cologne, Germany; European Vascular Center Aachen Maastricht, Department of Vascular Surgery, University Hospital RWTH Aachen, Aachen, Germany

**Keywords:** aortic, ARCH, repair, endovascular

## Abstract

**Objectives:**

The gold standard for the treatment of pathologies of the aortic arch remains the open surgical reconstruction of the affected segments. However, endovas-cular treatment options have emerged that eliminate the need for invasive open surgery. Several endograft devices – with fenestrations or branches for the supraaortic vessels – are currently available to address different pathologies and anatomical variations. Parallel-graft techniques and *in situ *fenestrations expand the treatment options for emergent cases. In this selective review of the literature of 2020 and 2021, we summarize the current chances and challenges of endovascular aortic repair.

**Content:**

Reported mortality rates range from 0 to 13.2 %. Although technical success rates for fenestrated and branched devices are promising (98 %), stroke rates remain a relevant issue (10 and 3 % for BTEVAR and FTEVAR respectively). The reported technical success rate for *in situ* fenestrations is also encouraging (94 %) and the stroke rates acceptable (5 %). Parallel-graft techniques are associated with high early and late endoleak rates (early 76 %; late 31 %), but still hold a valuable place in the treatment of emergent cases or in bail-out situations.

**Summary and Outlook:**

The endovascular repair of the aortic arch expands the range of patients with pathologies of the arch eligible for treatment to those unfit for open surgery offering a minimally invasive, yet technically challenging procedure. More data and meta-analyses are needed to define the benefits and drawbacks of this promising treatment option in an aging population with increasing co-morbidities.

## Introduction

Pathologies of the aortic arch, such as aortic dissections, aneurysms and penetrating aortic ulcers, may present themselves in acute and chronic form. The acute onset of symptoms in the setting of an aortic dissection involving the aortic arch (type A of the Stanford classification) demand immediate surgical attention and treatment in order to attenuate the risk of fatal complications – free rupture, pericardial tamponade or occlusion of the coronary arteries. In the same notion, ruptured aneurysms of the aortic arch are also life-threatening entities requiring emergent surgical treatment.

Currently, the gold standard of the surgical treatment of these acute pathologies is open surgery. The choice of the appropriate operative modality is dependent on the presentation of the pathology. After sternotomy and initiation of the cardio-pulmonal bypass (CBP), in most cases the ascending aorta is replaced with a graft and, in case of a concomitant valvular defect, the aortic valve is also replaced or reconstructed. The distal end of the reconstruction may lie before the ostium of the brachiocephalic trunk or extend in the aortic arch, which in this case is either partially or completely replaced with the graft. Supra-aortic vessels are then re-implanted in the reconstruction separately or as an island-patch. In cases of dissections or aneurysms involving the descending aorta as well, a further procedure has gained momentum in current clinical practice: the frozen-elephant trunk. This hybrid procedure introduces a stent–graft in the descending aorta, which is proximally fixed on the graft used for the open reconstruction of the ascending aorta and the aortic arch. In case of pathologies extending in the thoracoabdominal aorta, the reconstruction can be followed by a second stage TEVAR.

Chronic cases of aneurysmatic degeneration of the aortic arch, with or without residual dissection, are monitored regularly, to detect aneurysm expansion or progression of the disease and timely indicate surgical treatment. Traditionally, the treatment of the chronic form of aortic arch pathologies is similar to the acute setting: open surgery. However, despite progressive procedural improvements in open surgical repair of the aortic arch, it remains highly invasive in its nature with a reported operative mortality of 5.3 % and neurological deficits of 3.4 % [1]. Further risks of the procedure are associated with the use of CBP and hypothermic circulatory arrest as well as exposure to transfusion products. In this notion, older patients and those with relevant comorbidities are considered high-risk surgical candidates or even unfit for open surgery.

Given the ground-breaking advantages of endovascular treatment of the descending aorta, which has been established as first choice treatment for pathologies distally of the left subclavian artery, several devices have been designed to extend applicability of the endovascular treatment in the aortic arch, eliminating in this way the need of median sternotomy and aortic clamping. There is a multitude of endovascular devices intended for the aortic arch, that are currently used in investigational device exemption programs. Endografts with fenestrations or branches present various designs – from single-branch devices to triple-branched endografts. These custom-made endografts require, however, long production times. *In situ* fenestrations and parallel-graft techniques are viable off-the-self options for emergent cases and for the bail-out situations in case of unintended coverage of the target supra-aortic vessels.

Early results of endovascular treatment modalities for the aortic arch are promising, considering the higher age and co-morbidities of the patients included in the endovascular cohorts of studies comparing endovascular treatments and open surgery. The rates of early mortality are ranging from 0 to 13.2 %. Neurological complications remain a relevant challenge with stroke rates up to 20 % and spinal cord ischemia rates up to 3.1 % [1]. Cerebrovascular thromboembolic insults may occur during manipulation of the devices in the aneurysmatic aorta and due to clamping and handling of the carotid arteries during retrograde puncture and cannulation. Furthermore, coverage of the segmental arteries may also impair spinal cord perfusion and lead to temporary or permanent paraplegia.

Technical success rates are subject to a learning curve. Late results in experienced centres report successful device deployment in 84.2–100 % of the cases [1]. Failure to completely exclude the aneurysm may occur by unsuccessful cannulation of the target vessels, endoleaks and unsuccessful passage of the large calibre devices through the iliac arteries, often due to kinking and small calibre of the iliac axis. Furthermore, the large profiles of the currently available devices – 20 to 25 French [2] – require large femoral bore holes, which may increase the rate of access complications. Although, mortality seems unaffected by the learning curve, secondary procedures and intraoperative complications are reported more often in the first treated cases.

A further technical challenge of the endovascular therapy of the aortic arch is securing an adequate proximal landing zone. Both the physiological, three-dimensional curvature of the aortic bow anatomy and the relatively short distance between the aortic valve and the ostium of the brachiocephalic trunk may limit the applicability of the endovascular devices. In cases of previous reconstructions of the ascending aorta, the graft provides in most cases a sufficient landing zone for proximal fixation, given that the graft is long enough, without kinking.

In this article we summarized in a non-systematic review, some of the literature of the past two years concerning the endovascular treatment modalities for the aortic arch, as this new territory is dawning with a promise to provide new alternatives for the treatment of patients with aortic arch pathologies.

## Methods

A research of literature of the years 2021 and 2022 on aortic arch repair was performed via PubMed. A total number of around 10 papers were to be selected for this article. Meta-analyses, multi centre prospective and retrospective trials, single centre retrospective analyses, systematic reviews were included. As a result a total number of 971 patients with pathologies of the aortic arch were identified in 11 studies.

**Table:** 

Author	Study type	Procedure	N of patients	Technical success	30-day mortality	FUP (months)	Survival	Target vessel patency	Freedom from reintervention
Hauck, S. R. et al. 2022	Feasibility CT-scan	b/f-TEVAR	101	96 %	–	–	–	–	–
Dueppers, P. et al. 2022	Singlecenter, retrospective	PG-TEVAR	33	37 %	9 %	48± 31	34 %	–	68 %
Li, Y. et al. 2021	Meta-analysis	*In situ* fenstration	117	94 %	11 % (MAE)	1–55	–	–	–
Lu, H., L. C. Huang, and L. W. Chen 2022	Singlecenter, retrospective	b/f/ch-TEVAR, *in situ*-fen	62	100 %	6.45 %	3.51	–	100 %	–
Stana, J. et al. 2021	Review	b/f/ch-TEVAR, *in situ*-fen, hybrid, OR	–	–	–	–	–	–	–
Hauck, S. R. et al. 2022	Multicenter, retrospective	b-TEVAR vs. f-TEVAR	20 vs. 54	95 % vs. 82 %	10 % vs. 0 %	31 vs. 40	35 % vs. 32 %	95 % vs. 100 %	95 % vs. 85 %
Nana, P. et al. 2022	Review	b/f-TEVAR customized and non-customized	2135	98,7 vs 98,3	3,8 % vs 5,4 %	24 vs 27	90,3 % vs 81,3 %	–	91 % vs 90,4 %
Czerny, M. et al. 2021	Multicenter, retrospective	b-TEVAR (Terumo)	43	–	9 % (in-hospital)	16±18	–	–	–
Liang, N. L. et al. 2022	Multicenter, prospective	1× b-TEVAR (Gore)	40	–	–	36	84 %	95 %	97 %
Brown, J. A. et al. 2021	Review	b/f/ch-TEVAR, *in situ*-fen	–	–	–	–	–	–	–
Schenning, R. C. and R. Al-Hakim 2021	Review	b/f/ch-TEVAR	–	–	–	–	–	–	–

## Literature review

### Parallel stent–grafts

Dedicated devices for the aortic arch are custom made products, manufactured specifically to negotiate the anatomic requirements of the patient they are intended for. Yet, these highly individualized grafts require considerable production times of several weeks, currently limiting their application to elective procedures. In emergency settings, clinicians may consider using *in situ* fenestration devices or parallel stent–graft techniques – “chimneys” or “periscopes” – which can be deployed off-the-self, but might compromise technical success rate and freedom from reintervention.

Although, parallel stent–grafting of the abdominal aorta has been widely replaced by fenestrated and branched devices, it still has a place in the aortic arch in the form of parallel-graft TEVAR (PG-TEVAR). The principle of PG-TEVAR – similar to the “chimney” and “periscope” techniques of the abdominal aorta-lies in the deployment of a covered stent–graft in the target vessel, which runs parallel to the main stent–graft in the aorta. In the case of “chimney”-grafts, the stent–grafts are delivered retrogradely after distal exposure of the target vessel. “Periscope”-grafts are deployed through femoral access [3]. “Chimneys” are often used for the revascularization of the brachiocephalic trunk and the left common carotid artery, whereas “periscopes” or “snorkels” are mostly intended for revascularization of the left subclavian artery.

The main concern of PG-TEVAR is the high rate of gutter-related, type Ia endoleaks, often resulting in progression of disease and secondary interventions. The incidence of postoperative type I endoleak has been reported to be as high as 76 % and late endoleak rate up to 31 % [4]. The reported incidence rates of type I endoleaks vary greatly in the literature, as they are influenced by many factors, including centre experience. In order to optimize sealing and reduce the gutter space, several techniques have been proposed, such as oversizing of the stent–grafts (the excess material would wrap around the parallel-grafts, reducing gutter space) and adequate long sealing zones. Yet, the long-term results of PG-TEVAR remain discouraging, with a reported rate of aneurysm-related mortality of 42.3 % in 5 years and freedom from reintervention in 47.2 % of the cases in 5 years [3] reserving PG-TEVAR rather for bail-out situations and emergency settings. Since PG-TEVAR is often used as a bail-out procedure outcome may be influenced by selection bias.

### *In situ* fenestration

*In situ* fenestration techniques provide *in vivo* customization options for endograft deployment in the aortic arch and maintaining blood flow in the supra-aortic vessels. The technique was first introduced in 2004 by McWilliams et al. and has since been further developed and optimized. The key advantages of these procedures are the independency of the fenestration from the proximal landing zone of the endograft and the off-the-self applicability of the devices, making the technique an attractive treatment modality for urgent cases [1, 3]. In comparison to PG-TEVAR, *in situ* fenestration demonstrates significantly lower endoleak rates (1.7 vs. 19.3 %) [5]. The reported technical success rates (94 %) and acceptable stroke rates (5 %) as described in a recent meta-analysis from Li et al. encourage the adoption of the procedure also for patients planed for elective surgery [5].

For the fenestration manoeuvres distal exposure and puncture of the target vessels is necessary. A challenging part of the procedure is maintaining cerebral perfusion during bilateral common carotid artery cannulation. Some centres achieve this through extra-corporeal bypass conduits, anastomosed distally of the puncture site [3], temporary femoral–carotid bypass [6] or placing a sheath from the right common artery to the ascending aorta, creating a gutter space for cerebral perfusion [6]. After placing the endograft in the intended position, the fenestration device is securely placed with a steerable sheath at the ostium of the target vessel, in order to minimize the time between branch occlusion and fenestration. Under rapid pacing of the right ventricle the endograft is deployed. Then, the sheath with the fenestration catheter is gently pressed orthogonially to the endograft and the fenestration is made. A guide wire is introduced in the hole, and it is subsequently enlarged with a balloon-catheter, followed by the deployment of the branch stent–graft, which can be gently dilated.

The methods used for *in situ* fenestrations can be divided into two categories: mechanical (needle or the tail of a guide wire) and energy-based (laser and radiofrequency fenestration). The material of the endograft plays an important role in the choice of the fenestration method, since different materials demonstrate different characteristics in their structure and compatibility with the fenestration modalities: multifilament polyethylene terephthalate (PET) grafts can be used for all fenestration methods, whereas radiofrequency fenestration can be less successful in polytetrafluethylene (PTFE) grafts [5]. Furthermore, tolerating the expansion of the fenestration hole with a balloon catheter and accommodating the target vessel stent may compromise the structural integrity of the endograft and lead to endoleaks.

### Fenestrated and branched

The demand for devices specifically designed for use in the aortic arch was met by the industry with the implementation of a variety of endografts like branched and/or fenestrated devices both custom-made and available of-the-shelf as well as scaffolds and others. Whereas fenestrated grafts are best suited to treat pathologies involving the inner curvature of the aortic arch due to an otherwise inadequate proximal sealing-zone branched endografts are suitable to treat most lesions that can also be treated with a fenestrated graft but further expand the range of applicability to lesions of the outer curvature and those involving the ishimaru-zone 0 but consequently making the procedure more challenging.

In order for an endovascular repair to be feasible an adequate proximal landing zone of over 25 mm should be present as well as a distance from the most distal coronary artery to the innominate artery of over 50 mm should be present in order to achieve adequate proximal apposition of the graft with the aortic wall as reported in the literature [7]. A maximal diameter of 38 mm at the level of the proximal aorta, the fenestrations/branches and the distal aorta is necessary for an oversizing of 15 % to be achieved thus preventing proximal endoleak and migration and/or endograft collapse [7]. The aortic arch should not be angled over 70° and there should not be extensive thrombus at the inner curvature resulting in a risk of bird-beak and endograft collapse. A diameter of 20 mm for the innominate artery should not be exceeded and a stenosis of the internal carotid arteries of over 70 % NASCET should be evaluated for staged or simultaneous treatment to reduce the risk of stroke. Finally a life-expectancy of over 2 years should be present when evaluating an endovascular repair [7].

Implantation of a fenestrated stent graft for aortic arch repair usually is performed via a transfemoral and right brachial or axillary artery access in order to maintain alignment of the fenestration with the target vessels via a through and through guidewire without the necessity to place bridging stents in the fenestrations. Some devices are pre-loaded by means of simplifying through and through access.

With branched devices femoral access as well as bilateral access of either the brachial or axillary artery or both common carotid arteries is required. Before the deployment of the branched device a bypass between the left common carotid and subclavian artery is performed either as part of a staged approach with time between the bypass and the BTEVAR or simultaneously. After implantation of the bypass the device is placed in the proximal ascending aorta and the branches are cannulated on the left side either via the carotido subclavian bypass or the left common carotid artery after a cut-down and on the right side either via the subclavian artery or the right common carotid artery followed by the deployment of the bridging stents and placement of a plug in the proximal left subclavian artery in case of a significant type 2 endoleak.

Customized branched or fenestrated grafts specifically designed to fit the patients anatomy allow for the highest reported technical success rates with 98.3 % for FTEVAR and 98.7 % for BTEVAR compared to non CMD’s with 76–94 % [8, 9]. Although technical success rates for customized endografts are satisfying stroke rates remain an issue with 12.3 % for FTEVAR and 11.0 % for BTEVAR [9]. Due to a manufacturing time of several weeks CMD’s are typically used for elective treatment of aortic arch pathologies.

Stroke appears to be more likely to occur with BTEVAR than with FTEVAR resulting from the increased mandatory manipulation in the common carotid arteries in BTEVAR compared to FTEVAR procedures. A study comparing two of-the-shelf devices reported a stroke rate of 10 vs. 3 % for BTEVAR and FTEVAR respectively [8].

Robust long term outcome data of FTEVAR and BTEVAR are pending jet mid-term outcomes for different devices appear to be satisfying with a reported overall-mortality of about 16–20 % and an aortic related mortality of 3 % within 16–18 months [10, 11].

## Conclusions

The endovascular repair of the aortic arch expands the range of patients with pathologies of the arch eligible for treatment to those unfit for open surgery offering a minimally invasive yet technically challenging procedure. Despite the ongoing improvements of the devices and techniques to treat the aortic arch with, stroke and pending data for long-term efficacy remain challenging factors and open surgery remains the gold standard. Therefor patient selection, respecting the anatomical requirements, a high-volume centre and an interdisciplinary approach is key to minimize complications and achieve satisfying results by identifying the patients with high chance of profiting form an endovascular treatment. More data and meta-analyses are needed to implement guidelines for this promising treatment option in an aging population with increasing co-morbidities.
